# Does exposure to the food environment differ by socioeconomic position? Comparing area-based and person-centred metrics in the Fenland Study, UK

**DOI:** 10.1186/s12942-017-0106-8

**Published:** 2017-09-06

**Authors:** Eva R. Maguire, Thomas Burgoine, Tarra L. Penney, Nita G. Forouhi, Pablo Monsivais

**Affiliations:** 1 UKCRC Centre for Diet and Activity Research (CEDAR), MRC Epidemiology Unit, University of Cambridge School of Clinical Medicine, Institute of Metabolic Science, Cambridge Biomedical Campus, Cambridge, CB2 0QQ UK; 2 MRC Epidemiology Unit, University of Cambridge School of Clinical Medicine, Institute of Metabolic Science, Cambridge Biomedical Campus, Cambridge, CB2 0QQ UK; 3 Department of Nutrition and Exercise Physiology, Washington State University Elson S Floyd College of Medicine, Spokane, WA 99210 USA

**Keywords:** Diet inequalities, Food environment, Fast food, Supermarkets, Socioeconomic status, Area deprivation, Geographic information systems, Density, Proximity, Food access

## Abstract

**Background:**

Retail food environments (foodscapes) are a recognised determinant of eating behaviours and may contribute to inequalities in diet. However, findings from studies measuring socioeconomic inequality in the foodscape have been mixed, which may be due to methodological differences. The aim of this cross-sectional study was to compare exposure to the foodscape by socioeconomic position using different measures, to test whether the presence, direction or amplitude of differences was sensitive to the choice of foodscape metric or socioeconomic indicator.

**Methods:**

A sample of 10,429 adults aged 30–64 years with valid home address data were obtained from the Fenland Study, UK. Of this sample, 7270 participants also had valid work location data. The sample was linked to data on food outlets obtained from local government records. Foodscape metrics included count, density and proximity of takeaway outlets and supermarkets, and the percentage of takeaway outlets relative to all food outlets. Exposure metrics were area-based (lower super output areas), and person-centred (proximity to nearest; Euclidean and Network buffers at 800 m, 1 km, and 1 mile). Person-centred buffers were constructed using home and work locations. Socioeconomic status was measured at the area-level (2010 Index of Multiple Deprivation) and the individual-level (highest educational attainment; equivalised household income). Participants were classified into socioeconomic groups and average exposures estimated. Results were analysed using the statistical and percent differences between the highest and lowest socioeconomic groups.

**Results:**

In area-based measures, the most deprived areas contained higher takeaway outlet densities (*p* < 0.001). However, in person-centred metrics lower socioeconomic status was associated with lower exposure to takeaway outlets and supermarkets (all home-based exposures *p* < 0.001) and socioeconomic differences were greatest at the smallest buffer sizes. Socioeconomic differences in exposure was similar for home and combined home and work measures. Measuring takeaway exposure as a percentage of all outlets reversed the socioeconomic differences; the lowest socioeconomic groups had a higher percentage of takeaway outlets compared to the middle and highest groups (*p* < 0.001).

**Conclusions:**

We compared approaches to measuring socioeconomic variation in the foodscape and found that the association was sensitive to the metric used. In particular, the direction of association varied between area- and person-centred measures and between absolute and relative outlet measures. Studies need to consider the most appropriate measure for the research question, and may need to consider multiple measures as a single measure may be context dependent.

## Background

Socioeconomic disparities in diet quality have been identified [1–3] and likely contribute to inequalities in health outcomes including obesity, type 2 diabetes and cardiovascular disease [4–7]. Pervasive inequalities in diet have led researchers to seek modifiable determinants as potential targets of public health policies and interventions. The built environment, including the local retail food environment, or ‘foodscape’, may influence dietary behaviours and as a consequence contribute to rising levels of obesity, through shaping the context in which people make their food decisions [8–11]. The foodscape may be important for explaining socioeconomic disparities in diet, with some evidence of foodscapes varying by socioeconomic status (SES) [11, 12].

Much of the evidence on disparities in food retail access has centred on supermarkets and takeaway food (‘fast-food’) outlets. Specifically, supermarkets are seen to offer a range of affordable fresh produce for preparation at home [13, 14]. The ready-prepared hot foods typically served in takeaway outlets have been positively associated with energy and fat intakes [15], with frequent consumption associated with weight gain over time [16, 17]. In the United States (US) systematic reviews have identified consistent evidence of limited supermarket access and high takeaway food outlet access in low-income and racially-segregated neighbourhoods [18–20]. However, outside of the US, the picture has been more mixed. In the United Kingdom (UK), Canada and Australia, a limited number of studies have found more takeaway outlets in deprived areas [21–25], with one exception from Glasgow, Scotland [26]. No UK studies have identified socioeconomic disparities in the geographic accessibility of supermarkets [23, 27, 28]. In New Zealand, findings have shown that all food outlets are more prevalent in deprived areas [29]. These studies tend to use area-based measures of SES, limiting further association of these exposures to individual-level dietary behaviours and health outcomes. Socioeconomic differences in foodscape exposure based on individual level measures are therefore important to understand.

Furthermore, a number of studies have recognised the importance of considering a wider range of food outlets when assessing potential neighbourhood impacts on diet [30–34]. It may be that outlets offering both ‘healthy’ and ‘unhealthy’ options are commonly co-located, given food outlets tend to cluster in commercial areas [30]. Few previous studies have examined the socioeconomic differences in foodscape exposure using a relative measure of the retail food landscape [35, 36]. A recent Canadian study reported how more deprived neighbourhoods in Waterloo were more likely to have a less healthy food retail mix [36], however these findings were in contrast to previous Canadian research [35], may not reflect the wider international context, and were not based on person-centred estimates of either food access or indicators of SES.

The majority of foodscape studies use Geographic Information Systems (GIS), which is now a common tool in public health research [8]. However, a number of review articles have noted that methodological differences limit comparisons between food environment studies [8, 9, 12]. As a result, such reviews have called for greater consistency across methods used, as well as consideration of specific aspects of the methods, when assessing associations between measures of the foodscape and both SES and other outcomes including diet and obesity.

The purpose of this study was to compare socioeconomic differences in foodscape exposure using a number of commonly-used GIS-based metrics to better understand the implications of selecting different metrics. Using a cohort sample of UK adults, we compared area-based (‘ecological’) and person-centred (‘egocentric’) methods. Moreover, as much of the person-centred methods published in the literature is based on exposures around the residence only, we analysed person-centred metrics derived from both residential exposures and combined home- and work-based exposures. We also examined both absolute measures of takeaway outlet and supermarket exposure and relative measures of takeaway outlet exposure across three socioeconomic indicators.

## Methods

### Study sample

The Fenland Study is a population-based, observational cross-sectional cohort study, which aims to understand the genetic, behavioural and environmental factors relating to obesity and diabetes in adults. Eligible participants were men and women born between the years 1950 and 1975, aged 30–62 years at recruitment, who were registered at participating general practices across Fenland and East Cambridgeshire. Exclusion criteria were diagnosed diabetes, psychotic or terminal illness, pregnancy and being unable to walk unaided. Recruitment ran from 2005 to 2014 [37].

At the time of requesting data for this study, data were available for 11,857 Fenland Study participants. Participant home and work postcodes were geocoded using GeoConvert [38], and mapped using a GIS software package (ArcMAP 10, ESRI). UK postcodes contain 15 addresses on average, and therefore represent addresses with relative precision [39]. Participants whose home postcodes were missing (n = 1276), incomplete or invalid (n = 121), or outside the study area (n = 31) were removed, leaving an analytic sample of 10,429 with complete home address data. For work-based exposure assessments, we further excluded those not in work (n = 963) or where work status data was missing (n = 94), as well as those missing work postcodes, who provided incomplete work postcodes, or whose work address was outside the study area (n = 2102), resulting in a sub-sample of 7270.

### Food outlet data

Food outlet data collection and classification for use alongside Fenland Study data has been previously reported [40]. Briefly, food outlet data were collected in November and December 2011 from ten local councils covering the study area. All food outlet owners in the UK are required to register their premises with local Environmental Health departments, and to notify the department if they are closing. Local councils therefore hold records of food outlets that are regularly updated [41]. These are considered the most accurate source of food outlet data in the UK [42]. For this study we focussed on the locations of takeaways, supermarkets, convenience stores, restaurants, and cafés, which together account for the majority of household food shopping and out-of-home eating [40].

### Foodscape exposure metrics

Two broad and commonly employed approaches to assessing the foodscape were compared [43–45]: area-based metrics and person-centred (individual-level) metrics. Area-based metrics define neighbourhoods using administrative boundaries, which in this study were lower super output areas (LSOAs) designed to contain an average of 1500 residents and 650 households [46]. There were 801 LSOAs within the Fenland Study area. When using area-based metrics, outlet counts are generally standardised by resident population to give a density measure of exposure. In this study, takeaway and supermarket outlet counts were standardised against mid-2011 LSOA population estimates [47].

Person-centred metrics capture a neighbourhood specific to the individual, typically centred on a study participants’ home location. In addition to home, we considered the workplace an important daily anchor point and therefore also assessed the inclusion of workplace exposure to capture wider exposure to the foodscape [48]. We counted numbers of food outlets within Euclidean (straight-line) buffers and street network buffers, which measure distance along the road network and thus account for land use, for the reported home and work postcodes of Fenland Study participants. Buffers were constructed at three distances: 800, 1000, and 1609 m (1 mile), based on common precedent for their use in the published literature, wherein they have been theorised as ‘walkable’ for an average adult in 10–15 min [49–52]. In addition, person-centred exposure was also measured as proximity to the nearest takeaway outlet and supermarket, calculated as the shortest street network distance using ArcGIS Network Analyst.

### Socioeconomic indicators

Area-level SES was defined using the 2010 Index of Multiple Deprivation (IMD) [53]. IMD is a composite measure of deprivation calculated from indicators in seven domains: income; employment; health and disability; education skills and training; barriers to housing and other services; crime; living environment. IMD assigns relative scores to LSOAs, with higher scores reflecting greater deprivation. Fenland Study LSOAs ranged in score from 1.01 (least deprived) to 61.39 (most deprived), spanning the first and ninety-eighth centiles of all scores across England. LSOAs were divided into tertiles of deprivation based on their IMD score.

Two common indicators of individual-level SES were used: highest educational attainment and equivalised income. These data were provided by Fenland Study participants in a self-completed general questionnaire. Participants’ highest educational attainment was collapsed into three categories (‘Low’, includes compulsory school education and equivalent qualifications, typically completed at 16 years of age; ‘Medium’, includes academic or vocational qualifications gained during further education, such as those that allow university entry; ‘High’, degree-level or equivalent qualifications). To adjust for household size, annual household income was equivalised using the OECD’s modified equivalence scale [54]. Equivalised income was calculated for each participant from the midpoint of the available household income categories and collapsed into three bands (<£23,000; £23,000–£42,999; ≥£43,000).

As this was a complete case analysis, missing socioeconomic data reduced sample sizes in person-centred models that included educational attainment and equivalised income to 10,276 and 9617 respectively for home-based assessments, and to 7169 and 6774 respectively for combined home and work assessments.

### Data analysis

Average counts of, and distances to the nearest of each supermarket and takeaway were calculated across socioeconomic groups (highest educational attainment and household income groups for person-centred exposure metrics; tertiles of IMD scores for area-based metrics). Average proximity to the nearest supermarket and takeaway outlet from home was calculated for each socioeconomic group. Relative differences in exposures between the highest and lowest socioeconomic groups were also calculated. Takeaway density as a percentage of the sum of all food outlet types (takeaways, supermarkets, convenience stores, restaurants, cafés) was calculated. This calculation resulted in missing values where there were both no takeaway outlets and no other outlets, that is, both the numerator and denominator were zero. This was a complete case analysis, such that missing values resulted in list wise exclusion from respective analyses. For example, where person-based residential neighbourhood food environment exposures were derived in relation to participant educational attainment, we included all participants with data for education, home address, and associated covariates. Participants included in these analyses could have been lacking income data and work addresses. The number of missing values also varied according to the size of spatial buffer used, as detailed in Appendix Table 6. Linear tests for trends were used to test differences in means across socioeconomic groups, with *p* < 0.05 considered statistically significant. All analyses were conducted in Stata 13 (StataCorp., 2013).

### Sensitivity analyses

As every LSOA for the total Fenland Study geographic area was included in main area-based analyses (n = 801 LSOAs)—including both LSOAs where Fenland participants lived and those where they did not—there was a potential for bias. Thus, we conducted a sensitivity analysis of only the subsample of LSOAs where Fenland Study participants lived (n = 285 LSOAs).

## Results

### Sample characteristics

Characteristics of the analytic sample are provided in Table 1. Of the home-based sample, 89.9% reported being in work. The home and the combined home and work samples were similar across key age, sex, education and income profiles (Table 1).

**Table 1:** Descriptive statistics for the Fenland Study analytic sample (n = 10,429)

Variable	n	%	Missing n (%)
Female	5392	51.7	0
Age (years)	10,429	100	0
29–39	1652	15.8	
40–49	4338	41.6	
50–64	4439	42.6	
Educational attainment^a^	10,276	98.5	153 (1.5)
Low	2091	20.1	
Medium	4698	45.1	
High	3487	33.4	
Equivalised income^b^	9617	92.2	812 (7.8)
<£23,000	2372	22.7	
£23,000–£42,999	3991	38.3	
≥£43,000	3254	31.2	
Work address reported	7927	76.0	2502 (24.0)
Work status	10,335	99.1	94 (0.9)
In work^c^	9372	89.9	
Not in work^d^	963	9.2	

^a^Educational attainment: ‘Low’ indicates compulsory school education and corresponding qualifications; ‘Medium’ indicates further education academic or vocational qualifications; ‘High’ represents degree or higher qualifications

^b^Total household income equivalised using a version of the OECD’s modified equivalence scale

^c^Listed as working ‘full time’, ‘part time’, ‘obtained new job’

^d^Listed as ‘retired’, ‘keeping house’, ‘unemployed’, ‘sick leave’

### Area-based exposures

The population-adjusted density of takeaway outlets was greatest in the most deprived LSOAs (Table 2), with 76% fewer outlets in the least deprived tertile of areas compared to the most, and with a significant gradient across the deprivation tertiles (*p* < 0.001). There was no significant difference in area-based supermarket density by deprivation (*p* = 0.126). When measured as a percentage of all outlets, takeaway outlets made up 21.5% in the most deprived tertile compared to 12.4% in the least deprived tertile.

**Table 2:** Area-based takeaway outlet and supermarket densities (mean, 95% CI) per 10,000 population

LSOAs^a^	Takeaway outlets (n)	Supermarkets (n)	Takeaway outlets (% all outlets)^d^
Most deprived	9.4 (7.4, 11.5)	1.4 (1.0, 1.8)	21.5 (18.6, 24.4)
Medium	4.9 (3.6, 6.1)	1.0 (0.7, 1.3)	15.0 (12.1, 17.9)
Least deprived	2.3 (1.7, 2.8)	1.0 (0.7, 1.3)	12.4 (9.7, 15.2)
p-Trend^b^	<0.001	0.126	<0.001
% Difference^c^	-76	-29	-42

^a^Lower super output areas (n = 801) in tertiles of deprivation, defined using 2010 Index of Multiple Deprivation

^b^Test for trend based on linear regression with tertiles of deprivation treated as a continuous variable

^c^Percent difference computed as 100*((least deprived-most deprived)/most deprived)

^d^All food outlets = takeaways, supermarkets, convenience stores, restaurants, cafes

### Person-centred exposures

#### Comparing geographic boundaries for home-based exposures

Around the home, mean takeaway exposure was significantly and positively associated with both equivalised income and level of education at each scale of Euclidean and Network buffers (Table 3). Of the two socioeconomic indicators, educational attainment revealed larger differences in takeaway exposure than equivalised income. For example, within 1 mile Euclidean buffers, the percentage increase in takeaway outlets between the lowest and highest socioeconomic group was 56% for level of education and 17% for equivalised income. While absolute counts of outlets were greater in the larger buffers, relative socioeconomic differentials were greater at the smallest buffer sizes. Within Network buffers across level of education, for example, the difference in takeaway outlets at 800 m was 69% and at 1 mile was 49%. Outlet counts were greater in Euclidean than Network buffers. Proximity to the nearest takeaway was 7% shorter for the most educated participants than the least educated, which was significantly different (*p* = 0.012). There was no significant difference between equivalised income groups in proximity to the nearest takeaway outlet.

**Table 3:** Home-based takeaway exposure by groups of educational attainment and equivalised income

	Euclidean buffers (no. of outlets)			Network buffers (no. of outlets)			Proximity to nearest (km)
	800 m	1 km	1 mile	800 m	1 km	1 mile	Mean
*Educational attainment* ^a^ *(n* *=* *10,276)*							
Low	2.6 (2.4, 2.8)	3.8 (3.6, 4.1)	7.8 (7.4, 8.2)	1.3 (1.2, 1.4)	1.9 (1.8, 2.13)	4.7 (4.5, 5.0)	1.55 (1.50, 1.60)
Medium	2.5 (2.4, 2.6)	3.6 (3.4, 3.7)	7.0 (6.7, 7.3)	1.24 (1.2, 1.3)	1.9 (1.8, 2.0)	4.3 (4.1, 4.5)	1.64 (1.59, 1.69)
High	4.3 (4.1, 4.5)	6.1 (5.9, 6.4)	12.2 (11.7, 12.6)	2.2 (2.0, 2.3)	3.2 (3.1, 3.4)	7.0 (6.7, 7.3)	1.45 (1.39, 1.51)
*p* (Highest to lowest)	<0.001	<0.001	<0.001	<0.001	<0.001	<0.001	0.012
% Difference^c^	65	61	56	69	68	49	-7
*Equivalised income* ^b^ *(n* *=* *9617)*							
<£23,000	2.9 (2.7, 3.1)	4.2 (4.0, 4.5)	8.6 (8.2, 9.0)	1.5 (1.4, 1.6)	2.3 (2.1, 2.4)	5.1 (4.8, 5.4)	1.55 (1.48, 1.62)
£23,000–£42,999	3.0 (2.8, 3.1)	4.3 (4.1, 4.5)	8.3 (7.9, 8.6)	1.5 (1.4, 1.6)	2.3 (2.1, 2.4)	5.1 (4.8, 5.3)	1.58 (1.52, 1.63)
≥£43,000	3.6 (3.4, 3.8)	5.1 (4.8, 5.3)	10.1 (9.7, 10.6)	1.8 (1.6, 1.9)	2.6 (2.5, 2.8)	5.9 (5.6, 6.1)	1.52 (1.46, 1.58)
*p* (Highest to lowest)	<0.001	<0.001	<0.001	<0.001	<0.001	<0.001	0.915
% Difference^c^	24	21	17	20	13	16	-2

Mean values (95% confidence intervals), and statistical and percent difference between the lowest and highest socioeconomic groups

^a^Educational attainment: ‘Low’ indicates compulsory school education and corresponding qualifications; ‘Medium’ indicates further education academic or vocational qualifications; ‘High’ represents degree or higher qualifications

^b^Total household income equivalised using a version of the OECD’s modified equivalence scale

^c^Percent difference computed as 100*((Higher SES-Lower SES)/lower SES)

Results for supermarkets were similar (Table 4). Supermarket exposures were significantly and positively associated with both socioeconomic indicators, with educational attainment showing larger differentials than equivalised income. Within 1 mile Euclidean buffers, for example, between the lowest and highest socioeconomic group, the difference was 136% for level of education and 37% for equivalised income. The absolute outlet counts were greater in larger Euclidean and Network buffer sizes, and the relative differences were greater for smaller buffer sizes. Distances to the nearest supermarket were significantly shorter for highly-educated participants (23% shorter) compared to those least educated, and for higher income participants (9% shorter) compared to those in the lowest income group.

**Table 4:** Home-based supermarket exposure by groups of educational attainment and equivalised income

	Euclidean buffer counts (no. of outlets)			Network buffer counts (no. of outlets)			Proximity to nearest (km)
	800 m	1 km	1 mile	800 m	1 km	1 mile	Mean
*Educational attainment* ^a^ *(n* *=* *10,276)*							
Low	0.4 (0.4, 0.5)	0.7 (0.6, 0.7)	1.4 (1.4, 1.5)	0.2 (0.2, 0.2)	0.3 (0.2, 0.4)	1.7 (1.6, 1.8)	3.73 (3.59, 3.88)
Medium	0.5 (0.5, 0.5)	0.7 (0.7, 0.7)	1.5 (1.4, 1.5)	0.2 (0.2, 0.2)	0.3 (0.3, 0.4)	1.7 (1.6, 1.8)	3.90 (3.80, 3.99)
High	1.1 (1.0, 1.1)	1.6 (1.5, 1.6)	3.3 (3.2, 3.4)	0.5 (0.4, 0.5)	0.7 (0.7, 0.8)	3.8 (3.7, 4.0)	2.87 (2.76, 2.98)
*p* (Highest to lowest)	<0.001	<0.001	<0.001	<0.001	<0.001	<0.001	<0.001
% Difference^c^	1175	129	136	150	133	124	-23
*Equivalised income* ^b^ *(n* *=* *9617)*							
<£23,000	0.6 (0.5, 0.6)	0.8 (0.8, 0.9)	1.9 (1.8, 2.0)	0.3 (0.2, 0.3)	0.4 (0.4, 0.4)	2.2 (2.0, 2.3)	3.67 (3.54, 3.81)
£23,000–£42,999	0.6 (0.6, 0.7)	0.9 (0.9, 1.0)	2.0 (1.9, 2.1)	0.3 (0.3, 0.3)	0.5 (0.5, 0.5)	2.3 (2.2, 2.4)	3.60 (3.49, 3.71)
≥£43,000	0.8 (0.8, 0.9)	1.2 (1.1, 1.3)	2.6 (2.4, 2.7)	0.4 (0.3, 0.4)	0.6 (0.5, 0.6)	2.9 (2.8, 3.1)	3.34 (3.22, 3.46)
*p* (Highest to lowest)	<0.001	<0.001	<0.001	<0.001	<0.001	<0.001	<0.001
% Difference^c^	33	50	37	33	50	32	-9

Mean values (95% confidence intervals), and statistical and percent difference between the lowest and highest socioeconomic groups

^a^Educational attainment: ‘Low’ indicates compulsory school education and corresponding qualifications; ‘Medium’ indicates further education academic or vocational qualifications; ‘High’ represents degree or higher qualifications

^b^Total household income equivalised using a version of the OECD’s modified equivalence scale

^c^Percent difference computed as 100*((Higher SES-Lower SES)/lower SES)

#### Geographic boundaries and combined home and work exposures

With combined home and work exposures, there was a consistent association between level of education and takeaway exposure, which was positive and significant for all Euclidean buffers and for 1 mile Network buffers (Table 5). For example, within the 1 mile Network buffer highest educated participants had 16% greater takeaway exposure than lowest educated participants (*p* < 0.001). The association between equivalised income and takeaway exposure was positive and significant within 1 mile Euclidean and Network buffers, with a 11% and 7% difference between the highest and lowest income groups, respectively.

**Table 5:** Combined home and work exposure to takeaway outlets and supermarkets, by groups of educational attainment and equivalised income

	Euclidean buffer counts (no. of outlets)			Network buffer counts (no. of outlets)		
	800 m	1 km	1 mile	800 m	1 km	1 mile
*Takeaway outlets*						
Educational attainment^a^ (n = 7169)						
Low	7.86 (7.39, 8.32)	10.73 (10.16, 11.29)	20.22 (19.29, 21.14)	4.55 (4.23, 4.87)	6.29 (5.89, 6.69)	12.74 (12.09, 13.38)
Medium	7.01 (6.72, 7.29)	9.53 (9.18, 9.89)	18.05 (17.46, 18.64)	4.00 (3.80, 4.20)	5.54 (5.29, 5.79)	11.34 (10.93, 11.76)
High	8.49 (8.15, 8.83)	12.35 (11.90, 12.81)	25.14 (24.32, 25.95)	4.48 (4.27, 4.70)	6.62 (6.32, 6.91)	14.82 (14.27, 15.37)
*p* (Highest to lowest)	0.026	<0.001	<0.001	0.725	0.191	<0.001
% Difference^c^	8	15	24	-2	5	16
Equivalised income^b^ (n = 6774)						
<£23,000	7.73 (7.28, 8.17)	10.59 (10.04, 11.14)	20.31 (19.38, 21.24)	4.47 (4.17, 4.77)	6.23 (5.84, 6.61)	12.62 (11.98, 13.26)
£23,000−£42,999	7.51 (7.20, 7.82)	10.47 (10.06, 10.88)	20.18 (19.48, 20.87)	4.16 (3.95, 4.37)	5.89 (5.62, 6.16)	12.50 (12.02, 12.99)
≥£43,000	7.91 (7.57, 8.25)	11.28 (10.83, 11.72)	22.49 (21.71, 23.27)	4.25 (4.03, 4.47)	6.13 (5.84, 6.42)	13.46 (12.93, 13.98)
*p* (Highest to lowest)	0.510	0.057	<0.001	0.236	0.686	0.048
% Difference^c^	2	7	11	-5	-2	7
*Supermarkets*						
Educational attainment^a^ (n = 7169)						
Low	1.38 (1.30, 1.46)	1.94 (1.84, 2.05)	3.74 (3.54, 3.94)	0.75 (0.70, 0.81)	1.05 (0.98, 1.12)	3.22 (3.04, 3.39)
Medium	1.32 (1.27, 1.37)	1.86 (1.79, 1.94)	3.65 (3.51, 3.79)	0.71 (0.68, 0.75)	1.00 (0.96, 1.05)	3.10 (2.98, 3.23)
High	2.20 (2.11, 2.28)	3.23 (3.12, 3.35)	6.66 (6.42, 6.90)	1.07 (1.02, 1.12)	1.60 (1.53, 1.66)	5.90 (5.67, 6.12)
*p* (Highest to lowest)	<0.001	<0.001	<0.001	<0.001	<0.001	<0.001
% Difference^c^	59	67	78	45	52	83
Equivalised income^b^ (n = 6774)						
<£23,000	1.43 (1.35, 1.51)	2.08 (1.96, 2.19)	4.15 (3.92, 4.38)	0.77 (0.71, 0.82)	1.08 (1.01, 1.14)	3.63 (3.43, 3.83)
£23,000−£42,999	1.61 (1.55, 1.68)	2.32 (2.23, 2.42)	4.56 (4.37, 4.74)	0.84 (0.80, 0.89)	1.21 (1.15, 1.26)	3.96 (3.79, 4.13)
≥£43,000	1.87 (1.79, 1.94)	2.68 (2.57, 2.79)	5.47 (5.25, 5.69)	0.93 (0.88, 0.98)	1.37 (1.30, 1.43)	4.75 (4.55, 4.95)
*p* (Highest to lowest)	<0.001	<0.001	<0.001	<0.001	<0.001	<0.001
% Difference^c^	31	29	32	21	27	31

Mean values (95% confidence intervals), trend statistics and percent difference between the lowest and highest socioeconomic groups

^a^Educational attainment: ‘Low’ indicates compulsory school education and corresponding qualifications; ‘Medium’ indicates further education academic or vocational qualifications; ‘High’ represents degree or higher qualifications

^b^Total household income equivalised using a version of the OECD’s modified equivalence scale

^c^Percent difference computed as 100*((Higher SES-Lower SES)/lower SES)

Absolute takeaway outlet counts for the combined home and work exposures were greater than home-only exposures. For example, within the 800 m Euclidean buffer, combined home and work exposures were two to three times greater than home-based exposures alone. However, the relative differences between socioeconomic groups were smaller than for home-based exposures. In addition, the relative differences were greater in larger than smaller buffer sizes. Highest educated participants had an additional 8% of takeaway outlets within 800 m buffers and 24% within 1 mile Euclidean buffers. In common with home-based exposures, outlet counts were greater in Euclidean than Network buffers.

Results for combined home and work exposures were similar for supermarkets, although unlike takeaway outlets, there were significant and positive associations with both socioeconomic indicators at every Euclidean and Network buffer scale. Educational attainment showed larger differentials than equivalised income, with a relative difference at the 1 mile Euclidean buffer of 78% between lowest and highest level of education and 32% between lowest and highest equivalised income. Similar to takeaway outlets, absolute counts were two to three times greater compared to home-based exposures only. Further, the relative differences in supermarket exposures were greater at larger buffer sizes. For example, compared to lowest educated participants, highest educated participants had 59% greater numbers of supermarkets within 800 m Euclidean buffers and 78% more supermarkets within 1 mile.

#### Takeaway outlets as a percentage of all food outlets

**Fig. 1:**
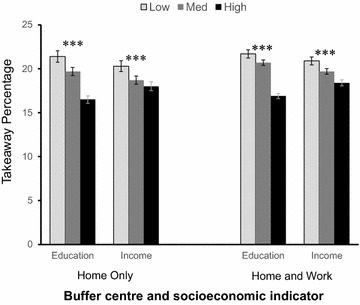
Takeaway outlets as a percentage of all outlets, 1 mile Euclidean buffers, home and combined home and work exposures. Mean values and error bars showing 95% confidence intervals. ***Linear test for trend across socioeconomic groups significant at the <0.001 level. ^a^ Educational attainment (‘education’): low, compulsory schooling; medium, further academic or vocational qualifications; high, degree or higher; Equivalised household income (‘income’): low, <£23,000; medium, £23,000–£42,999; high, ≥£43,000

Takeaway exposure as a percentage of all outlets was negatively and significantly associated with both indicators of individual SES. This association was found for both home and combined home and work exposures. For example, in the 1 mile home-based Euclidean buffer (Fig. 1), takeaway outlets constituted 21.4% of all outlets for the least educated and 16.5% of all outlets for the most educated. There was a smaller but significant difference between low income (20.3%) and high income (18.0%) groups. Results were similar for combined home and work exposure showing that takeaway outlets comprised 21.7% of all outlets for the least educated participants compared to 16.9% for the most educated, and 20.9% for the lowest compared to 18.4% for the highest income participants.

Similar results were found for all other Euclidean and Network buffers (Appendix Table 6). Average home-based and combined home and work-based exposures by SES to convenience stores, restaurants, cafés and all outlets are also shown in Appendix Table 7.

**Table 6:** Home and combined home and work exposures (mean, 95% CI) to takeaway outlets as a percentage of all food outlets

	Home			Home and Work		
	800 m	1 km	1 mile	800 m	1 km	1 mile
*Educational attainment* ^*a*^						
Euclidean	N = 9276^d^	N = 9568^d^	N = 9929^d^	N = 6890^d^	N = 6961^d^	N = 7067^d^
Low	22.2 (21.2, 23.2)	23 (22.1, 23.9)	21.4 (20.8, 22.1)	21.1 (20.4, 21.8)	21.7 (21.1, 22.3)	21.7 (21.2, 22.2)
Medium	20.0 (19.4, 20.7)	19.9 (19.4, 20.5)	19.7 (19.2, 20.1)	20.2 (19.8, 20.7)	20.5 (20.1, 20.9)	20.7 (20.4, 21)
High	18.1 (17.5, 18.7)	17.9 (17.4, 18.4)	16.5 (16, 16.9)	17.1 (16.6, 17.5)	17.0 (16.6, 17.3)	16.9 (16.6, 17.2)
p-trend^c^	<0.001	<0.001	<0.001	<0.001	<0.001	<0.001
*Network*	N = 7850^d^	N = 8717^d^	N = 9592^d^	N = 6479^d^	N = 6769^d^	N = 7002^d^
Low	20.4 (19.1, 21.8)	22.2 (21.3, 23)	21.9 (21.4, 22.5)	20.5 (19.6, 21.4)	20.8 (20.1, 21.6)	21.3 (20.8, 21.8)
Medium	18.4 (17.5, 19.2)	20.3 (19.7, 20.8)	20.5 (20.1, 20.9)	19.1 (18.5, 19.7)	19.7 (19.2, 20.2)	20.2 (19.9, 20.6)
High	16.8 (16.1, 17.5)	17 (16.5, 17.5)	16.6 (16.2, 16.9)	15.9 (15.4, 16.5)	16.5 (16, 17)	16.4 (16.1, 16.7)
p-trend^c^	<0.001	<0.001	<0.001	<0.001	<0.001	<0.001
*Equivalised income* ^*b*^						
Euclidean	N = 8,609^d^	N = 8,856^d^	N = 9,189^d^	N = 6,524^d^	N = 6,588^d^	N = 6,686^d^
<£23,000	21.3 (20.4, 22.2)	21.7 (20.9, 22.5)	20.3 (19.7, 21)	20.3 (19.6, 20.9)	20.8 (20.3, 21.4)	20.9 (20.4, 21.3)
£23,000–£42,999	19.5 (18.9, 20.2)	19.6 (19, 20.2)	18.7 (18.3, 19.2)	19.5 (19, 20)	19.6 (19.2, 20)	19.7 (19.3, 20)
≥£43,000	19.1 (18.4, 19.8)	18.9 (18.3, 19.5)	18.0 (17.5, 18.5)	18.4 (17.9, 18.9)	18.3 (17.9, 18.8)	18.4 (18, 18.7)
p-trend^c^	<0.001	<0.001	<0.001	<0.001	<0.001	<0.001
Network	N = 7498^d^	N = 8281^d^	N = 9014^d^	N = 6153^d^	N = 6424^d^	N = 6627^d^
<£23,000	18.7 (17.5, 19.9)	21.0 (20.2, 21.7)	21 (20.5, 21.5)	19.3 (18.4, 20.1)	19.8 (19.1, 20.6)	20.4 (19.9, 20.9)
£23,000−£42,999	18.2 (17.4, 19.1)	19.6 (19, 20.1)	19.4 (19, 19.8)	18.5 (17.9, 19.1)	19 (18.4, 19.5)	19.2 (18.8, 19.5)
≥£43,000	17.8 (16.9, 18.7)	18.1 (17.6, 18.7)	18.2 (17.8, 18.6)	17.2 (16.5, 17.8)	17.7 (17.1, 18.2)	17.9 (17.5, 18.3)
p-trend^c^	0.071	<0.001	<0.001	<0.001	<0.001	<0.001

All food outlets = takeaways, supermarkets, convenience stores, restaurants, cafes

^a^Educational attainment: ‘Low’ indicates compulsory school education and corresponding qualifications; ‘Medium’ indicates further education academic or vocational qualifications; ‘High’ represents degree or higher qualifications

^b^Total household income equivalised using a version of the OECD’s modified equivalence scale

^c^Test for trend based on linear regression with groups of education/income treated as a continuous variable

^d^Analytic sample size restricted to person-centred neighbourhoods containing a minimum of one none takeaway food outlet

**Table 7:** Home-based exposures (mean, 95% CI) to convenience stores, restaurants, cafés and all outlets, Euclidean and Network 1 mile buffers

Home	Convenience stores		Restaurants		Cafés		All outlets	
	1 mile euclidean	1 mile network	1 mile euclidean	1 mile network	1 mile euclidean	1 mile network	1 mile euclidean	1 mile network
Educational attainment^a^ (n = 10,276)								
Low	9.0 (8.5, 9.5)	5.3 (5.0, 5.6)	10.2 (9.5, 11.0)	5.6 (5.2, 6.0)	5.8 (5.3, 6.3)	3.0 (2.6, 3.3)	34.3 (32.3, 36.3)	20.2 (19.0, 21.5)
Medium	8.0 (7.7, 8.2)	4.8 (4.6, 4.9)	10.7 (10.1, 11.3)	5.9 (5.6, 6.3)	6.4 (6.0, 6.8)	3.3 (3.0, 3.5)	33.5 (32.0, 35.0)	19.9 (19.0, 20.8)
High	13.3 (12.8, 13.7)	7.5 (7.2, 7.8)	30.1 (28.7, 31.5)	15.7 (14.9, 16.6)	20.0 (19.0, 20.9)	9.8 (9.2, 10.4)	78.8 (75.4, 82.2)	43.8 (41.7, 45.9)
p-trend^c^	<0.001	<0.001	<0.001	<0.001	<0.001	<0.001	<0.001	<0.001
% Difference^d^	48	42	195	180	245	227	129	117
Equivalised income^b^ (n = 9617)								
<£23,000	9.9 (9.5, 10.4)	5.8 (5.5, 6.1)	14.2 (13.1, 15.2)	7.6 (7.0, 8.2)	8.5 (7.8, 9.2)	4.2 (3.8, 4.7)	43.0 (40.4, 45.7)	24.9 (23.2, 26.5)
£23,000−£42,999	9.4 (9.0, 9.7)	5.6 (5.3, 5.8)	15.7 (14.8, 16.6)	8.5 (8.0, 9.1)	9.6 (9.0, 10.3)	4.8 (4.5, 5.2)	44.9 (42.6, 47.2)	26.3 (24.9, 27.7)
≥£43,000	11.2 (10.7, 11.6)	6.3 (6.1, 6.6)	22.0 (20.8, 23.3)	11.4 (10.7, 12.2)	14.3 (13.4, 15.1)	6.9 (6.4, 7.5)	60.2 (57.2, 63.2)	33.5 (31.7, 35.3)
p-trend^c^	<0.001	<0.001	<0.001	<0.001	<0.001	<0.001	<0.001	<0.001
% Difference^d^	13	9	55	50	68	64	50	35
*Home and work* ^*e*^								
Educational attainment^a^ (n = 7169)								
Low	23.0 (21.9, 24.1)	14.2 (13.5, 15.0)	32.6 (30.4, 34.8)	21.0 (19.4, 22.6)	19.3 (17.8, 20.8)	12.7 (11.5, 13.8)	98.8 (93.4, 104.2)	63.8 (59.9, 67.7)
Medium	20.3 (19.6, 21.0)	12.6 (12.1, 13.1)	33.2 (31.6, 34.8)	21.4 (20.3, 22.5)	20.4 (19.3, 21.4)	13.2 (12.4, 14.0)	95.5 (91.7, 99.3)	61.7 (59.0, 64.4)
High	27.7 (26.8, 28.6)	16.2 (15.6, 16.7)	69.1 (66.2, 72.0)	42.7 (40.6, 44.7)	45.3 (43.3, 47.2)	27.9 (26.5, 29.3)	173.9 (167.3, 180.5)	107.4 (102.8, 112.1)
p-trend^c^	<0.001	<0.001	<0.001	<0.001	<0.001	<0.001	<0.001	<0.001
% Difference^d^	20	14	112	103	135	120	76	68
Equivalised income^b^ (n = 6774)								
<£23,000	23.0 (21.9, 24.1)	14.2 (13.5, 14.9)	36.6 (34.1, 39.2)	22.9 (21.1, 24.6)	22.3 (20.6, 24.0)	14.0 (12.8, 15.2)	106.4 (100.3, 112.5)	67.3 (63.1, 71.4)
£23,000−£42,999	22.7 (21.9, 23.5)	13.9 (13.3, 14.4)	43.2 (41.1, 45.4)	27.5 (26.0, 29.1)	26.9 (25.4, 28.3)	17.2 (16.1, 18.3)	117.5 (112.6, 122.5)	75.1 (71.5, 78.6)
≥£43,000	25.1 (24.2, 26.0)	14.9 (14.3, 15.5)	55.2 (52.6, 57.9)	34.4 (32.6, 36.2)	35.5 (33.8, 37.3)	22.1 (20.9, 23.4)	143.8 (137.8, 149.9)	89.6 (85.4, 93.8)
p-trend^c^	0.001	0.084	<0.001	<0.001	<0.001	<0.001	<0.001	<0.001
% Difference^d^	9	5	51	50	59	58	35	33

^a^Educational attainment: ‘Low’ indicates compulsory school education and corresponding qualifications; ‘Medium’ indicates further education academic or vocational qualifications; ‘High’ represents degree or higher qualifications

^b^Total household income equivalised using a version of the OECD’s modified equivalence scale

^c^Test for trend based on linear regression with groups of education/income treated as a continuous variable

^d^Percent difference is of the additional exposure in the highest socioeconomic group compared to the lowest

^e^Participants included if they had a valid work postcode and were in work

All food outlets = takeaways, supermarkets, convenience stores, restaurants, cafes

### Sensitivity analysis

In sensitivity analyses, we compared these results, which were derived from analyses of all LSOAs (n = 801) within the extent of the Fenland Study area, to those obtained from analyses which only included LSOAs (n = 285) containing Fenland Study participants. These alternate results (not shown) were not materially different from those presented here.

## Discussion

### Summary of findings

We compared the socioeconomic differences in foodscape exposure across a number of GIS-based metrics, and found that the direction and magnitude of socioeconomic differences was metric dependent. Using an area-based metric, takeaway outlets were more concentrated in more deprived areas. This is consistent with the majority of existing area-based work across international contexts [21, 22, 24, 25, 55, 56]. Area-based analysis of supermarkets showed no association between density and area deprivation, consistent with findings from previous UK-based research [23, 27, 28].

**Fig. 2:**
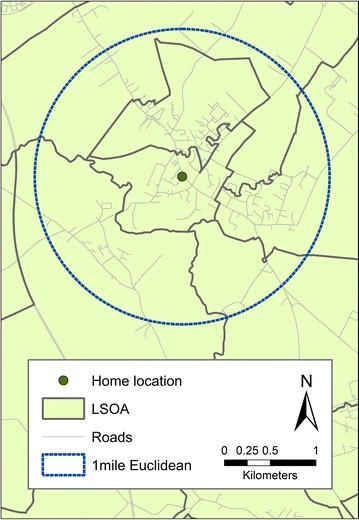
Comparison of lower super output area (area-based) boundaries and 1 mile Euclidean (person-centred) buffer. ^©^Crown Copyright and Database Right 2015. Ordnance Survey (Digimap Licence)

The most notable finding in this study is the contrast between the area-based and person-centred measures, when person-centred exposure was characterised as counts of outlets. The area-based results provided evidence that deprived areas have more takeaway outlets, and numbers of supermarkets equivalent to those found in less deprived areas, whereas using person-centred measures, takeaway and supermarket counts were greater for those with higher levels of education and income. There are multiple potential explanations for this discordance. Firstly, an individual’s own SES (based on income or educational attainment) may not be aligned with the SES of their assigned ‘neighbourhood’ (i.e. the deprivation level of their LSOA of residence) [57], or even their specific location *within* their assigned neighbourhood. There will inevitably exist unmeasured variation in levels of deprivation especially within and across larger administrative areas, such that these estimates cannot be attributed confidently to all residents [58]. Secondly, administrative boundaries such as LSOAs were not designed to capture the extent of their resident’s movements [44, 59], in the way that our person-based metrics of exposure are a better (although imperfect) approximation [60]. For smaller administrative boundaries in particular, individuals’ ‘activity spaces’ (defined as “the sub-set of all locations within which an individual has direct contact as a result of his or her day-to-day activities” [61]) are likely to extend beyond their limits [62], potentially resulting in foodscape exposure that is not reflected in estimates based solely on their assigned residential LSOA (Fig 2). These activity spaces would also likely be heterogeneous in socioeconomic conditions, with the individual exposed to neighborhoods socioeconomically disimilar to their own. This uncertain geographic context problem [63] may partly explain why socioeconomic differentials in absolute person-based food exposure are attenuated when accounting for food outlets around the work place.

By contrast, relative takeaway outlet exposure (takeaways as a percentage of all outlets), which better accounts for wider food environment context, showed the same (negative) socioeconomic gradient in both person- and area-level analyses, and this relationship was not attenuated by the addition of work-based exposure estimates. This relative approach has precedent [31, 33–36, 64–68], and has the potential to minimise residual confounding in analyses relating food outlet exposure to health [55]. In addition, relative access may be a more important conceptualisation of the impact of food outlet exposure on dietary behaviours than absolute numbers [12]. For example, while a high concentration of takeaway outlets offering a range of takeaway options might encourage more frequent consumption, the salience of this exposure may be reduced in the presence of retailers who are offering healthier food options [33]. Prior studies have associated higher relative takeaway outlet densities with low neighbourhood SES [36, 65], higher weight status [33, 34, 64, 67], and lower diet quality [68]. Our findings support the hypothesis that absolute counts of individual outlet types do not provide a complete picture of foodscape exposures.

**Fig. 3:**
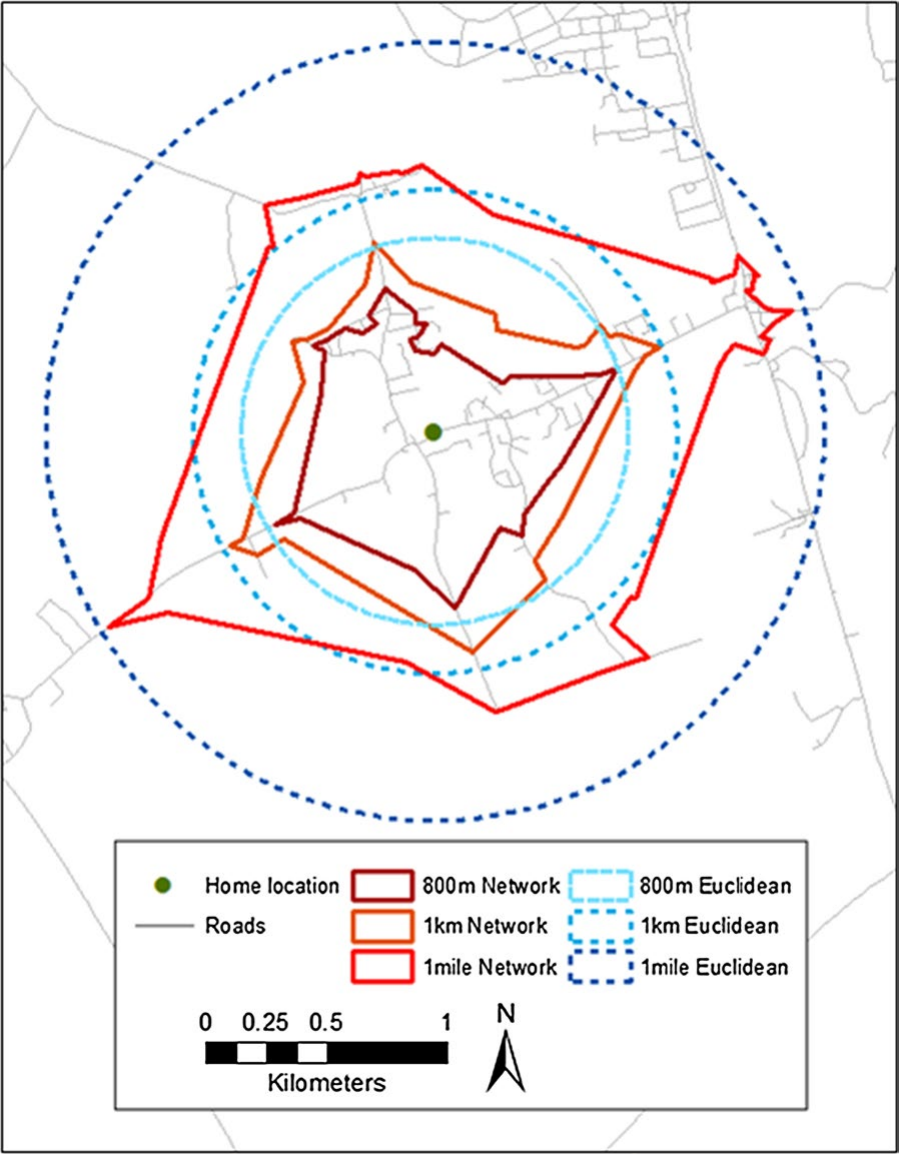
Comparison of person-centred euclidean and network buffers (800 m, 1 km, 1 mile). ^©^Crown Copyright and Database Right 2015. Ordnance Survey (Digimap Licence)

Our neighbourhood foodscape exposure estimates also showed sensitivity to buffer scale and type specifications, emerging from what has been described in the geographical literature the modifiable areal unit problem’s (MAUP’s) ‘scale’ and ‘zonation’ effects [69]. As illustrated in Fig. 3, irrespective of buffer type larger buffers tend to contain greater numbers of food outlets, resulting in greater estimates of exposure to the foodscape. Similarly, at any given scale, network buffers (that account for land use and may be considered more realistic delineators of neighbourhood than Euclidean buffers [70] generally have a smaller footprint than Euclidean equivalents (Fig. 3), and are therefore likely to contain fewer outlets, resulting in reduced estimates of foodscape exposure. The MAUP is a common consideration in the statistical analysis of geographical data, but has received limited attention in the food environments literature, wherein exposure estimates driven by methodological choices may reveal differential associations with individual-level outcomes of interest. Importantly however, the relative differences between socioeconomic groups in outlet counts remained present across all combinations of buffer scale and type, and were particularly similar across buffers of equal size irrespective of type (i.e., little evidence of MAUP zonation effect was observed when determining differences in outlet densities across socioeconomic groups).

There was also some sensitivity in our findings across the two indicators of individual SES tested, with larger differences between the groups highest and lowest educated, than between those on highest and lowest incomes. This suggests that the foodscape is structured according to local residents’ education more so than their income, and may reflect a greater spatial segregation among participants on the basis of their educational background. Alternatively or in addition, it may also reflect the targeting of stores according to neighbourhood sociodemographics.

Finally, the food outlet proximity measures tested also showed evidence of socioeconomic difference. The observed consistency with density measures is consistent with the findings of previous work, which has shown a strong correlation in this regard [71]. However, while proximity measures are useful for assessing geographic access to the closest outlet, they do not address the wider issues of the concentration or mix of outlets in the neighbourhood, which may be important to consider.

### Implications for further research

Given that the choice of GIS metric and specifications thereof can influence results, it is important that studies using GIS-based measures of the foodscape are aware of whether and to what extent methodological choices matter, and to conduct sensitivity analyses accordingly. For example, the null associations observed among smaller Network buffers for combined home and work exposure to takeaway outlets suggests that at smaller scales, Type-II errors may be introduced, where an association that exists is not identified, an issue previously identified by Thornton et al. [52]. The choice of metric should be informed by theories of the expected mechanisms by which the foodscape influences diet, and which features of the foodscape are seen as important. For example, takeaway outlets are increasingly seen as particularly relevant within the context of the work ‘neighbourhood’, with behavioural evidence from a US study showing that trips to fast-food outlets occur frequently on workdays [72]. However, given the time constraints of breaks at work, it may be that smaller buffer sizes are more appropriate in this context, in order to capture where workers are able to travel for food purchases, when examining the potential influence of takeaway outlets around the workplace [40]. On the other hand, the appropriate scale for assessing supermarket access may be larger given evidence that shoppers travel longer distances for grocery shopping trips [72], and that they are less likely to use their local supermarket if it is not also economically accessible to them [73]. Proximity measures could inform the size of spatial buffers to capture specific outlet types. Here, the median distance from residents to takeaway outlets was less than 1 km and to supermarkets was up to 2 km. Studies may use such distances to specify exposures by outlet type, as seen in a recent paper examining restaurant and supermarket exposures in relation to dietary behaviours and body mass index [32].

### Methodological considerations and limitations

This study is not without limitations. Some degree of temporal mismatch is possible, as the Fenland Study data collection ran from 2005 to 2014 and the food outlet data were collected in 2011. As relative SES is understood as a fairly stable construct [74], the level of error from changes in SES should be minimal. However inaccuracies may still result from any moves in participants’ home or work location. Further, addressing food environment exposure around the workplace does not provide a full assessment of exposure within broader activity spaces. While GIS techniques allow proxy measures, recording the extent of people’s actual activity spaces is possible with global positioning systems (GPS) technologies, an approach that is gradually becoming more common [62, 75, 76]. In addition, participants who did not report being in work were excluded from this wider assessment, and their exposure restricted to the residential neighbourhood. This has potential implications for understanding socioeconomic differences in exposure, as those not in work for certain reasons (e.g. unemployed or on sick leave) may be more likely to be disadvantaged more generally, thus skewing the sample towards higher SES participants. However, there is some evidence that being out-of-work is predictive of having a smaller daily activity space [75], potentially resulting in more accurate approximations of activity space exposure to food outlets when only addressing residential neighbourhood exposure for this group. While 24% of our sample did not have valid work addresses, we found that this percentage did not vary by area deprivation (data not shown), suggesting the lack of this information did not bias the socioeconomic profile of the sample.

The Fenland Study area includes urban and rural areas, and as such is fairly typical of many regions of the United Kingdom. The spread of LSOAs in this sample across the first and ninety-eighth percentile of IMD scores for England, further suggests that our findings are generalisable. With regard to the individual SES measures, the proportions of study participants in the highest education and income groups were also similar to the national average. For example, in 2012/13, 40% of non-retired households in England had an equivalised income upwards of £39,000 [77], while in 2011, 30% of the population held a degree qualification [78]. However, our sample contained a smaller representation of individuals with lower income and less education. For example, in 2011, 13% of residents in the same region had no qualifications [78]. However, the underrepresentation of lower socioeconomic groups among study cohorts is a general concern not unique to our study [79]. Moreover, underrepresentation of low SES groups may only have served to underestimate the scale of inequalities in foodscape exposures between the highest and lowest income groups.

The study also has a number of strengths. Most importantly, we systematically assessed multiple commonly-used foodscape exposure metrics using a large study cohort with detailed sociodemographic information, allowing two indicators of individual-level SES to be used. In addition, the inclusion of workplace location in the cohort allows an important additional anchor point in people’s regular activity space to be included in the measured exposure. In measuring the foodscape, food outlet data were collected from a validated source [41], the accuracy of which is comparable to other secondary food outlet location datasets commonly utilised in food environments research [80].

## Conclusions

This study showed associations between socioeconomic status and absolute exposure to takeaway outlets and supermarkets, and relative exposure to takeaway outlets, around home and work locations in a large UK sample of working age adults. The study provides a reference to health researchers interested in measuring the socioeconomic differences in exposure to the food environment and the impact that the choice of metric can have on results. As such, the results presented here can inform further investigations of associations between SES and foodscape exposure , to explore the role that the neighbourhood retail food environment plays in dietary inequalities.

## Appendix

See Tables 6 and 7.

## Abbreviations

IMD: Index of Multiple Deprivation
GIS: geographical information systems
GPS: global positioning systems
LSOA: lower super output area
MAUP: modifiable areal unit problem
OECD: Organisation for Economic Co-operation and Development
SES: socioeconomic status
UK: United Kingdom
US: United States
